# Gravitational 3D Magnetic Resonance Elastography for Differentiating Focal Nodular Hyperplasia and Hepatic Adenoma

**DOI:** 10.3390/diagnostics16101569

**Published:** 2026-05-21

**Authors:** Leon David Gruenewald, Shayan Mansouri, Christian Booz, Jennifer Gotta, Philipp Reschke, Tommaso D’Angelo, Mohamed Alrahmoun, Scherwin Mahmoudi, Simon S. Martin, Katrin Eichler, Tatjana Gruber-Rouh, Stefan Zeuzem, Esra Görgülü, Melis Onay, Eva Herrmann, Maria Johanna Gobertina Tetuanui Vehreschild, Katharina Schregel, Sandra Ciesek, Sebastian Haberkorn, Thomas Joseph Vogl, Ralph Sinkus, Vitali Koch

**Affiliations:** 1 Clinic for Radiology and Nuclear Medicine, University Hospital, Goethe University Frankfurt, 60596 Frankfurt am Main, Germany; 2 Department of Internal Medicine I, University Hospital, Goethe University Frankfurt, 60596 Frankfurt am Main, Germany; 3 Institute of Biostatistics and Mathematical Modeling, Faculty of Medicine, Goethe University Frankfurt, 60596 Frankfurt am Main, Germany; 4 Department of Internal Medicine, Infectious Diseases, University Hospital, Goethe University Frankfurt, 60596 Frankfurt am Main, Germany; 5 Section Neuroradiology, Institute of Diagnostic and Interventional Radiology, University Hospital Jena, 07747 Jena, Germany; 6 Institute of Medical Virology, University Hospital, Goethe University Frankfurt, 60596 Frankfurt am Main, Germany; 7 Department of Cardiology and Angiology, University Hospital Frankfurt, 60596 Frankfurt am Main, Germany; 8 Team “From Micro to Macro in Cancer Development”, Center for Research on Inflammation, UMR 1149 National Institute for Health and Medical Research (INSERM), Université de Paris, 75013 Paris, France; ralph.sinkus@kcl.ac.uk; 9 School of Biomedical Engineering and Imaging Sciences, King’s College London, London WC2R 2LS, UK

**Keywords:** liver, magnetic resonance elastography, focal nodular hyperplasia, hepatic adenoma, elastography, shear wave speed, gravitational transducer, benign liver tumors

## Abstract

**Background/Objectives:** Differentiating focal nodular hyperplasia (FNH) from hepatic adenoma (HA) remains challenging, as FNH is benign whereas HA carries risks of hemorrhage and malignant transformation. This prospective single-center pilot study evaluated the diagnostic performance of three-dimensional magnetic resonance elastography (3D-MRE) using a gravitational transducer for non-invasive differentiation of FNH and HA. **Methods:** Thirty-three participants (23 FNH, 10 HA) underwent 3D-MRE using the gravitational transducer. Viscoelastic parameters—stiffness, shear wave speed (Cs), wave attenuation, and phase angle—were quantified for lesions and background parenchyma. Δ-values were calculated by subtracting background liver measurements from lesion values. **Results:** FNH demonstrated significantly higher stiffness than HA (median 3.16 vs. 2.58 kPa; *p* = 0.02) and higher Cs (median 1.81 vs. 1.64 m/s; *p* = 0.001). Normalized stiffness differences (Δ stiffness) were significantly greater in FNH than HA (median 0.83 vs. 0.10 kPa; *p* = 0.001). Generalized additive models revealed divergent volume-dependent stiffening behaviors. In ROC analysis, Δ stiffness and Δ Cs each achieved an AUC of 0.87, indicating that single background-normalized viscoelastic parameters carry the principal diagnostic signal. An exploratory multivariable combination of Δ stiffness with patient age produced an apparent AUC of 0.93 with wide odds-ratio confidence intervals, and is presented as hypothesis-generating rather than as a clinical prediction model. **Conclusions:** In this pilot cohort, 3D-MRE using the gravitational transducer showed encouraging parameter-level separation between FNH and HA, with background normalization enhancing discrimination. Wave attenuation and phase angle did not differ significantly between lesion types. Given the small sample size (particularly the HA subgroup of ten patients), the mixed reference standard (histological confirmation in only 14 of 33 lesions; definitive hepatobiliary-phase MRI criteria in 19 of 33), the single-slice ROI used for lesion measurement, and the incomplete characterization of background liver parenchyma, these findings should be regarded as hypothesis-generating and require external validation in larger, multicenter cohorts before any clinical application.

## 1. Introduction

Focal nodular hyperplasia (FNH) and hepatic adenoma (HA) are the most common benign solid liver tumors. Despite their benign classification, they present a diagnostic challenge with significant management implications. FNH is typically asymptomatic with no recognized risk of malignant transformation [1,2]. In contrast, HA is associated with hemorrhage and a risk of malignant transformation to hepatocellular carcinoma (HCC), particularly in lesions larger than 5 cm or in specific molecular subtypes, such as β-catenin-activated HCA. Consequently, management strategies diverge, from conservative surveillance for FNH to surgical intervention for HA [3].

While liver biopsy is considered the definitive standard for resolving diagnostic ambiguities, it is an invasive procedure with inherent limitations, including sampling error, inter-observer variability, and a risk of complications [4]. Non-invasive assessment relies on multiparametric magnetic resonance imaging (MRI) with hepatobiliary contrast agents [3,5]. However, FNH and HA frequently overlap in imaging features; for instance, specific subtypes, such as inflammatory hepatocellular adenoma (IHCA), may mimic FNH by appearing isointense in the hepatobiliary phase, leading to equivocal results in a clinically significant subset of cases [3,5].

Elastography offers a fundamentally different approach to tissue characterization by assessing biomechanical properties rather than contrast-uptake dynamics. Several prior studies have explored this concept for focal liver lesions. Venkatesh et al. provided early evidence that MR elastography (MRE) can quantify the stiffness of various liver tumors, though sample sizes for FNH and HA were limited [6]. Shahryari et al. subsequently demonstrated that multifrequency MRE-based tomoelastography can distinguish benign from malignant liver lesions with high accuracy based on tissue stiffness and fluidity [7]. Using ultrasound-based shear wave elastography (SWE), Brunel et al. reported that FNH was significantly stiffer than HA (mean 47.0 vs. 12.1 kPa; *p* < 0.001), supporting the diagnostic potential of biomechanical assessment [8]. However, Taimr et al. found that point SWE (pSWE) could not reliably differentiate FNH from HA due to significant variability and overlap in stiffness values, likely attributable to the limitations of single-point sampling [9]. These conflicting findings highlight the need for an elastographic approach with three-dimensional wave field acquisition, which may reduce sampling error compared with single-point measurements. Importantly, the clinical translation of quantitative elastography depends not only on diagnostic separation but equally on measurement reproducibility and acquisition standardization. Structured frameworks for assessing the repeatability and reproducibility of quantitative elastographic measurements, including intra-observer, inter-observer, intra-session, and inter-session agreement, have been established in ultrasound-based elastography [10,11,12,13], and such frameworks underscore the need for rigorous assessment of measurement reliability in any new elastographic technique.

MRE provides a larger sampling volume and greater tissue penetration than ultrasound elastography [14]. The fundamental principle involves an external active driver generating low-frequency mechanical shear waves (typically 60 Hz), which are imaged by a motion-sensitive MRI sequence [15]. From the resulting wave fields, quantitative stiffness maps can be generated [16]. Recent developments in this field include a novel gravitational transducer that uses an eccentrically rotating mass to generate mechanical vibrations. This technology may enhance the quality and accuracy of viscoelastic reconstructions compared to conventional acoustic driver systems, thereby facilitating more advanced tissue characterization; subsequent technical validation has confirmed superior signal-to-noise ratios and wave penetration for hepatic imaging [17,18].

We hypothesized that the distinct pathophysiological and histological features of FNH and HA result in measurably different biomechanical properties. The primary objective of this single-center pilot study was to evaluate the feasibility and preliminary diagnostic performance of three-dimensional MRE for the non-invasive differentiation of FNH from HA. The gravitational transducer served as the enabling hardware, whose technical validation and comparison with conventional acoustic drivers have been reported separately [17,18].

## 2. Materials and Methods

This prospective, cross-sectional, single-center study was approved by the institutional review board (No. 20-887, dated 13 October 2020) and conducted in accordance with the principles outlined in the Declaration of Helsinki. Written informed consent was obtained from all participants.

### 2.1. Patient Selection

**Figure 1:**
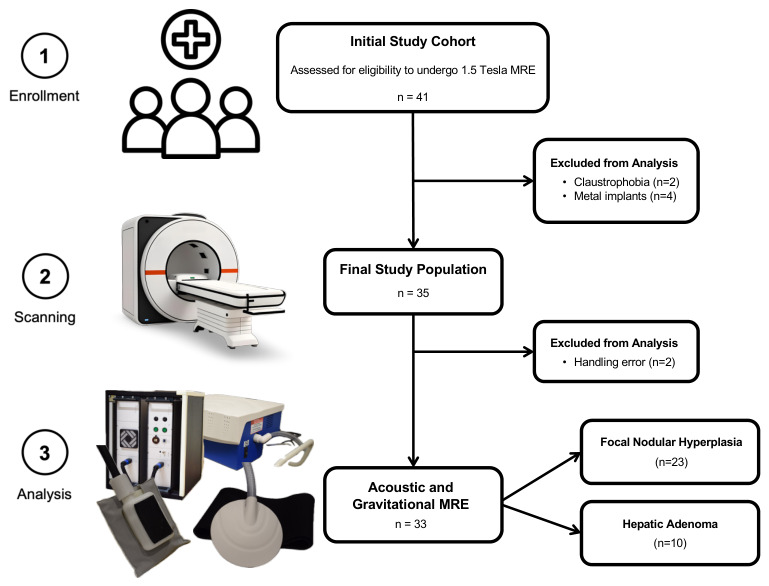
Study flowchart illustrating patient enrollment, exclusions, and final study population. Forty-one patients were initially assessed for eligibility to undergo 1.5 Tesla magnetic resonance elastography (MRE). After exclusion for claustrophobia and metal implants, 35 patients remained, of whom 33 underwent 3D-MRE using the gravitational transducer and were included in the final analysis.

Eligible patients were identified by prospectively querying digital health records and the institutional Picture Archiving and Communication System (PACS) for patients with focal liver lesions clinically suspected to be FNH or HA who were scheduled for either histological sampling or MRI with hepatospecific gadolinium-based contrast agents between September 2024 and October 2025. Patients with focal liver lesions suspected to represent other entities (e.g., hemangiomas, hepatocellular carcinoma, or metastases) were not enrolled. Exclusion criteria were: (a) prior locoregional or systemic therapies for the target lesion; (b) MRI examinations compromised by severe motion or susceptibility artifacts; and (c) incomplete health records. The final study population comprised 33 patients with 33 lesions: 23 had confirmed FNH and 10 had confirmed HA. Lesion confirmation was obtained through histological sampling in 14 patients and through definitive MRI criteria using hepatospecific gadolinium-based contrast agents in 19 patients (Figure 1). Definitive MRI diagnosis of FNH was based on the presence of a well-circumscribed lesion with arterial-phase hyperenhancement, absence of washout in the portal venous and delayed phases, iso- or hyperintensity on hepatobiliary-phase imaging, and, when present, a central scar with delayed enhancement. HA was diagnosed on MRI based on characteristic features, including arterial-phase hyperenhancement, absence of hepatobiliary-phase uptake, variable intralesional fat or hemorrhage, and lack of a central scar. When MRI findings did not meet these definitive criteria or remained inconclusive, histological confirmation by biopsy was performed. All 10 HA cases were histologically confirmed. In 6 cases, conventional MRI features were equivocal or atypical, including iso- to hyperintense signal on the hepatobiliary phase mimicking FNH, a recognized diagnostic pitfall associated with inflammatory and beta-catenin-activated HCA subtypes [19,20,21]. In the remaining 4 HA cases, imaging findings were characteristic, but a biopsy was performed to exclude malignancy. Among FNH, 4 of 23 (17%) had equivocal imaging findings necessitating histological confirmation; the remaining 19 were diagnosed based on definitive MRI criteria.

### 2.2. MR Imaging Protocol

MRE was employed to provide a non-invasive method for the quantitative assessment of tissue biomechanics. All MRE examinations were performed on a 1.5 T MRI scanner (Siemens Aera, Siemens Healthineers, Forchheim, Germany).

The entire clinical liver MRI protocol has been described previously [17]. It included an anatomical fat-saturated T1-weighted 3D gradient-echo Dixon VIBE (volumetric interpolated breath-hold examination), T2-weighted 2D turbo spin-echo, and diffusion-weighted 2D sequences. The gravitational transducer was positioned in a standardized manner along the mid-axillary line to align its main vibrational direction with the acquisition readout axis (i.e., in the right–left direction), thereby minimizing possible motion-related artifacts. The gravitational 3D-MRE acquisition employed the Ristretto scheme with fractional motion encoding (gradient-echo sequence, frequency 60 Hz, echo time [TE] = 9.53 ms, repetition time [TR] = 104.25 ms, field of view [FOV] = 384 mm, flip angle 25°), as described previously [22]. Parameters of the 3D-MRE protocol are listed in the Supplementary Material. Signal reception utilized an 18-channel body matrix coil and (parts of) a table-integrated 32-channel spine matrix coil. The gravitational MRE research demonstrator setup, including the external rack with electronics and a motor, the flexible shaft, and the gravitational transducer with its patient attachment and comfort features, has been described in detail previously [17].

### 2.3. Method for 3D-MRE Stiffness Reconstruction

**Figure 2:**
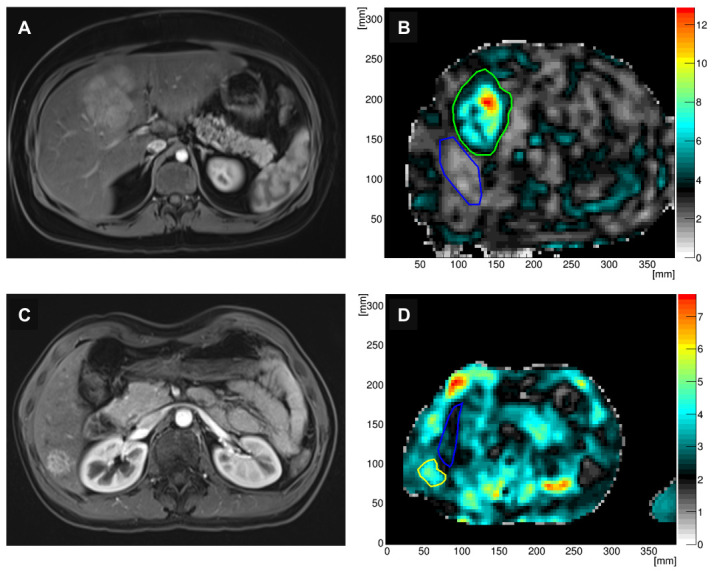
Representative magnetic resonance imaging and elastography findings in benign focal liver lesions. (**A**) Axial contrast-enhanced T1-weighted MR image in the hepatobiliary phase demonstrating a focal nodular hyperplasia. (**B**) Corresponding three-dimensional magnetic resonance elastography stiffness map showing elevated stiffness within the lesion (green ROI) relative to surrounding liver parenchyma (blue ROI). (**C**) Axial contrast-enhanced T1-weighted MR image of a hepatic adenoma. (**D**) Corresponding stiffness map with regions of interest placed within the lesion (green ROI) and adjacent liver tissue (blue ROI). Note the matched field of view and orientation between anatomical and parametric images. ROI, region of interest.

The reconstruction pipeline for gravitational 3D-MRE has been described in detail previously [17,23] and is summarized in the Supplementary Material. In brief, postprocessing of the 3D-MRE data involved application of a mathematical curl operator to suppress compressional wave components and isolate shear wave motion. The resulting shear wave fields were then analyzed by solving the Helmholtz equation to generate quantitative maps of the complex shear modulus ($G^{*}$). From these maps, the following viscoelastic parameters were derived: stiffness ($|G^{*}|$, kPa), shear wave speed (Cs, m/s), wave attenuation (1/m), and phase angle (π/2). Wave attenuation quantifies the spatial decay rate of shear waves and is inversely related to the mechanical wave penetration depth in tissue. Confidence and stiffness maps were generated offline using the research 3D reconstruction algorithms developed for this study. The term “3D-MRE” refers to the volumetric wave acquisition and the subsequent 3D reconstruction pipeline (curl filtering and Helmholtz inversion), not to volumetric ROI analysis. Regions of interest (ROIs) for gravitational MRE were delineated by a radiologist with seven years of experience in abdominal imaging (L.D.G.), who was blinded to clinical and histopathological details. Lesion ROIs were manually delineated on a single axial slice displaying the largest tumor cross-section on co-registered anatomical T1-weighted images (hepatobiliary or arterial phase), carefully excluding surrounding parenchyma. This single-slice ROI approach was chosen to standardize measurements and ensure clinical feasibility, while benefiting from the superior wave field quality afforded by the 3D acquisition. Background liver ROIs were placed in the right hepatic lobe parenchyma at the same or an adjacent slice level, excluding large vessels, liver margins, and deeper parenchymal regions prone to wave attenuation artifacts (Figure 2). ROI-based measurements were extracted from co-registered parametric maps.

### 2.4. Statistical Analysis

All statistical analyses were performed using Python (Version 3.11). Normality was assessed using the Shapiro–Wilk test. Variables are presented as mean ± standard deviation (SD) for normally distributed data or as median with interquartile range (IQR) for non-normally distributed data. Group differences were assessed using Welch’s *t*-test or the Mann–Whitney *U* test. A *p*-value less than 0.05 was regarded as statistically significant. Given the exploratory nature of this pilot study and the multiple comparisons performed across absolute and background-normalized parameters, no formal correction for multiple testing was applied. The reported *p*-values should therefore be interpreted as descriptive and hypothesis-generating rather than confirmatory.

Inter-reader reproducibility was assessed using intraclass correlation coefficients (ICCs; two-way random effects model, absolute agreement). ICC values were interpreted as: poor (<0.50), moderate (0.50–0.74), good (0.75–0.89), and excellent (≥0.90).

Multivariable logistic regression with recursive feature elimination was used to evaluate the ability of MRE parameters to differentiate between FNH and HA. Continuous variables were standardized (Z-transformed) before inclusion in the models. Diagnostic performance was quantified using the area under the receiver operating characteristic curve (AUC). Generalized additive models (GAMs) using penalized thin-plate regression splines were employed to visualize relationships between lesion volume and viscoelastic parameters. Internal validation of the logistic regression models was performed using bootstrap optimism correction (1000 iterations) and leave-one-out cross-validation (LOOCV). Model calibration was assessed using the Hosmer–Lemeshow goodness-of-fit test (5 groups) and the Brier score. Sensitivity and specificity 95% confidence intervals were calculated using the exact Clopper–Pearson method.

## 3. Results

### 3.1. Study Cohort and Baseline Lesion Characteristics

The study population comprised 33 patients (median age, 40 years [IQR, 31–49 years]; 26 females) with 33 benign liver lesions (23 FNH, 10 HA). Baseline characteristics are detailed in Table 1.

### 3.2. Baseline 3D Elastography Measurements

Inter-reader agreement for ROI-based viscoelastic measurements was excellent (ICC = 0.92, 95% CI: 0.88–0.96). FNH lesions demonstrated distinct viscoelastic properties compared to HA (Table 2). The median stiffness of FNHs was significantly higher than that of HA (3.16 kPa [IQR, 2.5–4.0 kPa] vs. 2.58 kPa [IQR, 2.2–2.8 kPa]; *p* = 0.02). Similarly, the median Cs was significantly higher in FNHs (1.81 m/s [IQR, 1.6–2.1 m/s]) compared to HA (1.64 m/s [IQR, 1.5–1.7 m/s]; *p* = 0.001). No significant differences were observed for wave attenuation (42.08 ± 11.30 1/m for FNH vs. 49.07 ± 13.75 1/m for HA; *p* = 0.18) and phase angle (0.26 ± 0.06 vs. 0.26 ± 0.05, respectively; *p* = 0.76). Representative examples of lesion and background liver ROI placement on anatomical images and corresponding stiffness maps are shown in Figure 2.

### 3.3. 3D Elastography Measurements Versus Background Liver

Background liver parenchyma measurements were obtained to normalize lesion values (Table 3). Mean background stiffness was 2.27 ± 0.35 kPa, mean Cs was 1.54 ± 0.12 m/s, mean wave attenuation was 57.67 ± 8.34 1/m, and mean phase angle was 0.29 ± 0.03. When normalized to background tissue, FNH lesions showed a significantly larger elevation in stiffness relative to the background (Δ stiffness median: 0.83 kPa [IQR: 0.4–1.9]) compared to HA (Δ stiffness median: 0.10 kPa [IQR: −0.1–0.3]; *p* = 0.001). The difference in Cs (Δ Cs) was also higher in FNH (0.37 ± 0.33 m/s) than in HA (−0.01 ± 0.16 m/s; *p* < 0.001). FNH lesions exhibited a greater reduction in wave attenuation relative to the background (Δ wave attenuation: −17.73 ± 15.81 1/m) compared to HA (−3.66 ± 11.56 1/m; *p* = 0.009). No significant difference was observed regarding the phase angle (*p* = 0.56).

### 3.4. Non-Linear Relationship Between Lesion Volume and Stiffness

**Figure 3:**
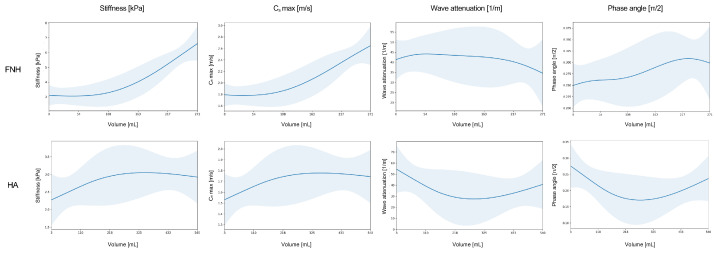
Volume-dependent behavior of viscoelastic parameters derived from magnetic resonance elastography in benign focal liver lesions. Smoothed regression curves (solid lines) with corresponding confidence intervals (shaded areas) illustrate stiffness (kPa), shear wave speed (Cs, m/s), wave attenuation (1/m), and phase angle (π/2) as a function of lesion volume. (**Upper row**): Focal nodular hyperplasia (FNH). (**Lower row**): Hepatic adenoma. Distinct volume-related trends are observed across all viscoelastic parameters for the two lesion types.

GAMs revealed distinct non-linear, volume-dependent stiffening behaviors. FNHs exhibited higher baseline stiffness than HA, with a monotonic increase at higher volumes. In contrast, HA demonstrated a slight linear increase in stiffness at lower volumes, which plateaued beyond approximately 250 mL, suggesting a saturation effect in which larger HA did not exceed a stiffness threshold of approximately 3.0 kPa. The divergence in stiffness between the two lesion types became more pronounced in larger lesions (Figure 3).

### 3.5. Diagnostic Performance

**Figure 4:**
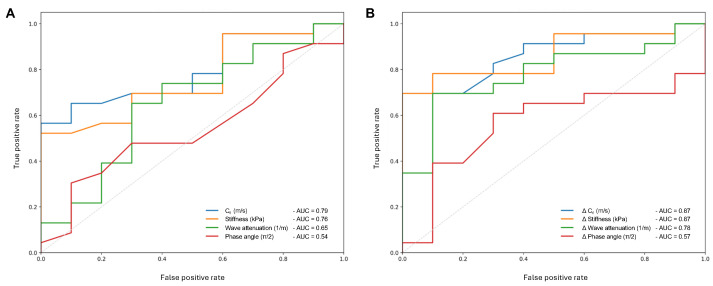
Receiver operating characteristic (ROC) analysis for differentiation of focal nodular hyperplasia and hepatic adenoma using magnetic resonance elastography-derived parameters. (**A**) Diagnostic performance of absolute viscoelastic parameters, including shear wave speed (Cs), stiffness, wave attenuation, and phase angle. (**B**) Diagnostic performance of background-normalized (Δ) parameters, including Δ stiffness, Δ Cs, Δ wave attenuation, and Δ phase angle. Area under the curve (AUC) values are indicated for each parameter. The dashed diagonal line represents chance-level discrimination.

ROC analysis was performed to establish optimal thresholds and diagnostic accuracy for both absolute metrics and difference-to-background parameters (Table 4, Figure 4). Among the absolute metrics, Cs demonstrated the strongest performance with an AUC of 0.79 (*p* = 0.009) and an optimal cutoff of 1.76 m/s, yielding 100% specificity but moderate sensitivity (57%). Absolute stiffness (cutoff: 3.16 kPa) achieved an AUC of 0.76 (*p* = 0.021). Normalizing measurements to the background liver significantly improved diagnostic performance. Both Δ stiffness (cutoff: 0.54 kPa) and Δ Cs (cutoff: 0.18 m/s) achieved the highest overall accuracy (AUC = 0.87; *p* = 0.001 and *p* < 0.001, respectively), with both metrics demonstrating 70% sensitivity and 100% specificity for distinguishing FNH from HA. Δ wave attenuation showed an AUC of 0.78 (*p* = 0.012), whereas the difference in phase angle, both absolute and relative, remained non-significant between lesions (AUC 0.54 and 0.57; *p* = 0.724 and *p* = 0.556, respectively).

### 3.6. Logistic Regression Models for Lesion Differentiation

Logistic regression models were developed to differentiate between FNH and HA using both absolute and Δ measurements. Due to collinearity among viscoelastic parameters, only one elastography metric was included per model. Recursive feature elimination resulted in a multivariate model that identified both absolute Cs (odds ratio [OR], 16.91; 95% confidence interval [CI]: 1.58–181.35; *p* = 0.02) and patient age (OR, 4.62; 95% CI: 1.27–16.76; *p* = 0.02) as significant independent predictors for differentiating FNH from HA. The overall AUC for this model was 0.91 (0.73–0.98). Through recursive feature elimination using Δ measurements, both the stiffness difference between the lesion and background parenchyma (Δ stiffness; OR, 24.61; 95% CI: 1.92–315.58; *p* = 0.01) and patient age (OR, 3.48; 95% CI: 1.00–12.12; *p* = 0.05) were found to be significant predictors. This model achieved an overall AUC of 0.93 (0.82–1.00) for differentiating the two lesion types (Table 5). The very wide odds-ratio confidence intervals for both models (for example, Δ stiffness OR 24.61, 95% CI 1.92–315.58; Cs OR 16.91, 95% CI 1.58–181.35) reflect instability of multivariable estimates after feature selection in a dataset of this size, and these models should therefore be regarded as exploratory and hypothesis-generating; the principal diagnostic signal of this study lies in the per-parameter separations reported above rather than in the combined model.

### 3.7. Internal Validation

Bootstrap optimism correction (1000 iterations) yielded optimism-corrected AUCs of 0.90 (Model 1) and 0.92 (Model 2), with a mean optimism of 0.01 for both models. LOOCV AUCs were 0.87 for both models. The Hosmer–Lemeshow test did not detect miscalibration for Model 1 ($\chi^{2}$ = 0.47; *p* = 0.92) or Model 2 ($\chi^{2}$ = 0.39; *p* = 0.94), and Brier scores were 0.11 and 0.10, respectively. Given the small sample size and the use of only five groups, the Hosmer–Lemeshow test has very limited statistical power in this setting; these results should therefore be interpreted as an absence of gross miscalibration rather than as strong evidence of well-calibrated probabilities, which would require assessment in an independent, adequately sized cohort.

### 3.8. Post Hoc Subgroup Analysis of Cases with Equivocal Imaging

To assess the diagnostic performance of 3D-MRE in diagnostically challenging cases, a post hoc subgroup analysis was performed on the 10 patients with equivocal or overlapping imaging features: 4 FNH cases with atypical imaging findings necessitating histological confirmation and 6 HA cases with equivocal or atypical imaging (e.g., iso- to hyperintense signal on the hepatobiliary phase mimicking FNH, or imaging features insufficient to confidently exclude malignancy). ROC analysis was performed for each viscoelastic parameter within this subgroup, with Youden’s J statistic used to determine optimal thresholds (Table 6).

Background-normalized parameters outperformed absolute values. Δ Cs achieved the highest AUC (0.90), and Δ wave attenuation demonstrated the best balanced accuracy (90%; sensitivity 100%, specificity 83%). The multivariable models (trained on the full cohort) correctly classified 9 of 10 (90%, Model 1) and 10 of 10 (100%, Model 2) equivocal cases at the standard probability threshold of ≥0.50. Because the ROC-derived thresholds for this subgroup were optimized within the same *n* = 10 cases, these figures reflect resubstitution performance in a very small, internally optimized subset and are highly vulnerable to chance; they should not be read as validation of diagnostic utility in equivocal imaging, but as a hypothesis-generating signal requiring prospective evaluation in an independent cohort of diagnostically challenging lesions.

## 4. Discussion

In this prospective single-center pilot study, we evaluated the feasibility of 3D-MRE using a gravitational transducer for differentiating FNH from HA. Our two main findings concern single-parameter behavior: first, FNH lesions are biomechanically stiffer and propagate shear waves faster than HA; second, background normalization (“Δ” measurements) improves per-parameter discrimination, with Δ stiffness and Δ Cs each achieving an AUC of 0.87. An exploratory multivariable combination of Δ stiffness with patient age produced an apparent AUC of 0.93, but with wide odds-ratio confidence intervals (for example, Δ stiffness OR 24.61, 95% CI 1.92–315.58; Cs OR 16.91, 95% CI 1.58–181.35) that reflect the limited sample size and reinforce that this combination is hypothesis-generating rather than a candidate clinical prediction model.

Our finding that FNH is stiffer than HA aligns with the known histopathology of these lesions. FNH is characterized by a central scar with radiating fibrotic septa and an abnormal arterial supply, creating a mechanically integrated fibrous framework that facilitates shear wave propagation; thus shear waves propagate faster. This confers increased stiffness scaling with lesion volume [24]. In contrast, HA consists predominantly of homogeneous hepatocyte plates with preserved sinusoidal architecture and minimal fibrotic reinforcement, leading to lower stiffness values and a plateauing stiffness–volume relationship [9,19]. This divergent volume-dependent behavior—monotonically increasing stiffness in FNH versus a plateau in HA—likely reflects the progressive accumulation of fibrotic scaffolding unique to the FNH architecture.

Our AUC of 0.87 for normalized stiffness exceeds the 0.67–0.69 reported by Taimr et al. using pSWE [9]. Several factors may contribute to this difference, including the three-dimensional wave field acquisition and curl-based reconstruction of 3D-MRE, which reduce sampling bias compared with single-point ultrasound measurements; the use of background normalization as an internal patient-specific control; and the inherently lower operator dependence of MRE [25]. The directional finding, FNH being stiffer than HA, is consistent across MRE [6,7] and SWE [8] modalities. Because both lesion types yield absolute stiffness values within the F0–F2 range of MRE-derived liver stiffness [26], normalization to background parenchyma is essential, and lesion-to-liver stiffness ratios have been shown to outperform absolute values for focal lesion differentiation [27]. We chose subtraction-based normalization (Δ values) because it preserves the original measurement units; ratio-based normalization yielded comparable performance in a post hoc analysis. Because the background liver was not formally characterized by fibrosis staging, proton density fat fraction, or R2* mapping, the interpretation of Δ values requires caution: subclinical differences in hepatic fibrosis, steatosis, or iron content between patients could influence baseline parenchymal stiffness and therefore the magnitude of Δ-based measurements.

The diagnostic performance observed here may be partly attributable to the three-dimensional wave field acquisition and reconstruction inherent to 3D-MRE. Compared with conventional 2D-MRE, the 3D approach offers three conceptual advantages for focal lesion characterization: (1) the curl operator isolates shear wave motion from compressional wave artifacts, which is not feasible in single-slice 2D acquisitions; (2) three-dimensional wave field coverage reduces partial-volume effects and sampling bias inherent to single-slice wave acquisition; and (3) multidirectional displacement encoding captures the full wave propagation field, yielding more robust estimates of the complex shear modulus. The gravitational transducer’s omnidirectional wave fields further support homogeneous coverage across hepatic segments [17,18]. It should be emphasized, however, that lesion ROIs in the present study were placed on a single representative slice for standardization and clinical feasibility; the measurements reported here therefore benefit from the three-dimensional wave field but do not constitute fully volumetric lesion characterization. A direct head-to-head comparison with 2D-MRE in the same cohort was not performed, so the incremental diagnostic value of the 3D approach over 2D-MRE cannot be quantified from our data.

Because MRE probes intrinsic mechanical tissue properties independent of hepatocyte transporter function, it provides a diagnostic axis that is conceptually orthogonal to hepatobiliary-phase MRI. The brief acquisition time (approximately four breath-holds totaling ∼56 s) would, in principle, allow integration into existing contrast-enhanced liver MRI protocols. Whether MRE-derived stiffness can ultimately strengthen diagnostic confidence for hypervascular lesions, or reduce the need for percutaneous biopsy, cannot be inferred from the present pilot cohort and must be addressed in dedicated prospective studies.

If confirmed in larger cohorts, 3D-MRE could conceivably serve as a second-line adjunct following conventional multiparametric MRI with hepatobiliary contrast agents, in particular for cases in which hepatobiliary-phase imaging yields equivocal findings, such as inflammatory or β-catenin-activated HA subtypes that mimic FNH. In our post hoc analysis restricted to the 10 cases with equivocal or overlapping imaging features, ROC analysis suggested that background-normalized parameters retained the diagnostic signal, with Δ Cs achieving an AUC of 0.90 and Δ wave attenuation yielding the highest balanced accuracy in this subgroup (90%; sensitivity 100%, specificity 83%). Because the ROC-derived thresholds were optimized within the same *n* = 10 cases, these figures represent resubstitution performance in a very small internally optimized subset and are highly vulnerable to chance; we therefore present them as a hypothesis-generating signal and not as evidence of diagnostic utility in equivocal imaging, which can only be assessed in an independent prospective cohort of diagnostically challenging lesions. The thresholds derived in this study (for example, Δ stiffness ≥ 0.54 kPa) were optimized within the present dataset and cannot be recommended for clinical use at this stage; their validity and potential role in treatment decisions, including any effect on biopsy indications, can only be assessed after external validation in independent cohorts. Before integration into clinical algorithms, several steps are required: (1) external validation in multicenter cohorts including diverse scanner platforms and HA molecular subtypes; (2) establishment of standardized acquisition and postprocessing protocols; (3) dedicated reproducibility studies assessing intra-observer, inter-observer, test–retest, inter-session, inter-scanner, and transducer-positioning variability, following established methodological frameworks for quantitative elastography [10,11]; (4) direct head-to-head comparison with 2D-MRE; and (5) evaluation of cost-effectiveness relative to established diagnostic pathways.

This study has several limitations. First, this single-center pilot study included a small cohort (*n* = 33), with only 10 HA cases, which limits statistical power and precision of the reported estimates, including those derived from internal validation. Although the modest optimism (∼1%) and stable LOOCV performance (AUC 0.87 for both models) suggest limited overfitting, the internally validated estimates cannot substitute for external validation, and the reported AUCs should therefore be considered apparent performance estimates rather than evidence of clinical readiness. Second, the reference standard was not uniformly histopathological: only 14 of 33 lesions (42%) were confirmed by biopsy, while 19 (58%) were diagnosed based on definitive hepatobiliary-phase MRI criteria using gadoxetic acid, which was specifically employed when prior imaging with conventional gadolinium-based contrast agents yielded equivocal results. Although hepatobiliary-phase imaging represents the current non-invasive reference standard for FNH versus HA differentiation, the partial reliance on imaging as a reference cannot be fully resolved retrospectively and introduces the risk of verification and classification bias, particularly for atypical HA subtypes that can mimic FNH on the hepatobiliary phase. Future prospective studies should aim for uniform histopathological confirmation of all included lesions. Third, lesion measurements were obtained from a single representative axial slice rather than from a full three-dimensional ROI; while this was deliberately chosen to standardize measurements and ensure clinical feasibility, it means that our results should not be interpreted as fully volumetric lesion characterization. Fourth, our reproducibility assessment was limited to inter-reader agreement on ROI placement (ICC = 0.92). Comprehensive reproducibility assessment of quantitative elastography, as established in ultrasound-based elastography, additionally requires intra-observer repeatability, within-session test–retest variability, between-session repeatability, inter-scanner variability, and an evaluation of the effect of transducer positioning on measurement consistency [10,11]. None of these were formally evaluated in the present study, and dedicated reproducibility studies will therefore be essential before gravitational 3D-MRE can be considered standardized for clinical use. Fifth, the background liver was not formally characterized: formal fibrosis staging was not performed, and proton density fat fraction and R2* maps were not acquired, so the interpretation of Δ parameters, which are central to our best-performing estimates, requires cautious reading. Sixth, from a biological and translational standpoint, we did not perform molecular subtyping of the HA cohort. HA is a heterogeneous entity whose subtypes may exhibit distinct stiffness profiles: steatotic HA may be softer due to fat content, whereas inflammatory subtypes may be stiffer [19,21,28]. Correlating viscoelastic signatures with molecular subtypes is a necessary next step, given that management is increasingly driven by genotype-specific risk stratification [29]. Seventh, a direct comparison with conventional 2D-MRE was not performed, limiting conclusions about the incremental value of the 3D approach. Finally, the exploratory nature of this study, with multiple comparisons across eight viscoelastic parameters, increases the risk of type I error inflation; the reported *p*-values should be interpreted accordingly, and the identified thresholds require prospective validation.

## 5. Conclusions

In this single-center pilot study of 33 patients, three-dimensional MRE using a gravitational transducer was feasible for the characterization of FNH and HA. At the level of individual viscoelastic parameters, background-normalized stiffness and shear wave speed separated FNH from HA with apparent AUCs of 0.87 each. An exploratory multivariable combination with patient age produced an apparent AUC of 0.93 (optimism-corrected 0.92, LOOCV 0.87) but with wide odds-ratio confidence intervals, and should be regarded as a hypothesis-generating signal rather than as a validated clinical prediction model. Given the limited sample size (particularly the small HA subgroup), the mixed reference standard, the single-slice ROI, the incomplete characterization of background liver parenchyma, and the absence of independent reproducibility, molecular, and multicenter data, these findings should be regarded as hypothesis-generating. The thresholds reported here were optimized within the present dataset and should not be applied clinically at this stage; external validation in larger multicenter cohorts, dedicated reproducibility studies, molecular characterization of HA subtypes, and direct comparison with 2D-MRE will be required before any conclusions can be drawn about the clinical role of this technique.

## Figures and Tables

**Table 1:** Baseline patient characteristics.

Variables	FNH (*n* = 23)	HA (*n* = 10)	*p*-Value
Age (years)	41 ± 11	36 ± 10	0.20
Sex			0.16
Male	3 (13%)	4 (40%)	–
Female	20 (87%)	6 (60%)	–
Lesion volume (cm^3^)	16.92 (7.3–48.3)	22.33 (6.9–61.2)	0.84
Liver stiffness (kPa)	2.18 ± 0.32	2.45 ± 0.35	0.05

Data are presented as mean ± standard deviation for normally distributed continuous variables, median (interquartile range) for non-normally distributed continuous variables, or *n* (%) for categorical variables. Between-group comparisons employed Welch’s *t*-test and the Mann–Whitney *U* test for continuous variables and Fisher’s exact test for categorical variables. FNH, focal nodular hyperplasia; HA, hepatocellular adenoma.

**Table 2:** Baseline 3D elastography lesion measurements across FNH and HA.

Variables	FNH (*n* = 23)	HA (*n* = 10)	*p* Value
Stiffness (kPa)	3.16 (2.5–4.0)	2.58 (2.2–2.8)	0.02
Cs (m/s)	1.81 (1.6–2.1)	1.64 (1.5–1.7)	0.001
Wave attenuation (1/m)	42.08 ± 11.30	49.07 ± 13.75	0.18
Phase angle (π/2)	0.26 ± 0.06	0.26 ± 0.05	0.76

Data are presented as median (interquartile range) following rejection of normality or mean ± standard deviation for normally distributed continuous variables. Between-group comparisons employed the Mann–Whitney *U* test and Welch’s *t*-test. FNH, focal nodular hyperplasia; HA, hepatocellular adenoma.

**Table 3:** Difference-to-background liver lesion measurements across FNH and HA.

Variables	FNH (*n* = 23)	HA (*n* = 10)	*p* Value	Background (*n* = 33)
Δ Stiffness (kPa)	0.83 (0.4–1.9)	0.10 (−0.1–0.3)	0.001	2.27 ± 0.35
Δ Cs (m/s)	0.37 ± 0.33	−0.01 ± 0.16	<0.001	1.54 ± 0.12
Δ Wave attenuation (1/m)	−17.73 ± 15.81	−3.66 ± 11.56	0.009	57.67 ± 8.34
Δ Phase angle (π/2)	−0.04 (−0.1–0.0)	−0.01 (−0.0–0.0)	0.56	0.29 ± 0.03

Data are presented as median (interquartile range) or mean ± standard deviation, depending on distribution. FNH, focal nodular hyperplasia; HA, hepatocellular adenoma.

**Table 4:** Receiver operating characteristic analysis results.

Variables	Threshold	Sensitivity	Specificity	PPV	NPV	AUC	*p* Value
**Absolute Values**							
Stiffness (kPa)	≥3.16	0.52 (0.31–0.73)	1.00 (0.69–1.00)	1.00	0.48	0.76 (0.59–0.90)	0.021
Cs (m/s)	≥1.76	0.57 (0.34–0.77)	1.00 (0.69–1.00)	1.00	0.50	0.79 (0.62–0.93)	0.009
Wave attenuation (1/m)	≤45.31	0.65 (0.43–0.84)	0.70 (0.35–0.93)	0.83	0.47	0.65 (0.41–0.86)	0.177
Phase angle (π/2)	≥0.30	0.30 (0.13–0.53)	0.90 (0.55–1.00)	0.88	0.36	0.54 (0.32–0.75)	0.724
**Δ Values**							
Δ Stiffness (kPa)	≥0.54	0.70 (0.47–0.87)	1.00 (0.69–1.00)	1.00	0.59	0.87 (0.72–0.97)	0.001
Δ Cs (m/s)	≥0.18	0.70 (0.47–0.87)	1.00 (0.69–1.00)	1.00	0.59	0.87 (0.73–0.97)	<0.001
Δ Wave attenuation (1/m)	≤−14.66	0.65 (0.43–0.84)	0.90 (0.55–1.00)	0.94	0.56	0.78 (0.60–0.93)	0.012
Δ Phase angle (π/2)	≤−0.03	0.61 (0.39–0.80)	0.70 (0.35–0.93)	0.82	0.44	0.57 (0.36–0.77)	0.556

Sensitivity and specificity 95% confidence intervals were calculated using the exact Clopper–Pearson method. Thresholds indicate the direction favoring FNH classification (≥ = values above threshold; ≤ = values below threshold). AUC, area under the curve; NPV, negative predictive value; PPV, positive predictive value; ROC, receiver operating characteristic; FNH, focal nodular hyperplasia; HA, hepatocellular adenoma.

**Table 5:** Logistic regression models for absolute and Δ variables.

Variables	Coefficient (95% CI)	Odds Ratio (95% CI)	AUC	*p* Value
**Model 1: Absolute Parameters**				
Cs (m/s)	2.83 (0.46–5.20)	16.91 (1.58–181.35)	0.91 (0.73–0.98)	0.02
Age	1.53 (0.24–2.82)	4.62 (1.27–16.76)	0.91 (0.73–0.98)	0.02
**Model 2: Background-Normalized (Δ) Parameters**				
Δ Stiffness (kPa)	3.20 (0.65–5.75)	24.61 (1.92–315.58)	0.93 (0.82–1.00)	0.01
Age	1.25 (−0.00–2.50)	3.48 (1.00–12.12)	0.93 (0.82–1.00)	0.05

Logistic regression analysis results for the differentiation of FNH from HA. Model 1 incorporates the shear wave speed (Cs) and age; Model 2 uses the stiffness difference (Δ stiffness) and age. All variables were standardized (Z-transformed) before analysis. *p*-values indicate the statistical significance of individual predictors within the multivariate models. AUC, area under the receiver operating characteristic curve; CI, confidence interval; FNH, focal nodular hyperplasia; HA, hepatocellular adenoma.

**Table 6:** ROC analysis in the equivocal imaging subgroup (*n* = 10).

Parameter	AUC	Threshold	Sensitivity	Specificity	Accuracy
Δ Cs (m/s)	0.90	≥0.04	4/4 (100%)	4/6 (67%)	8/10 (80%)
Δ Wave attenuation (1/m)	0.88	≤−6.22	4/4 (100%)	5/6 (83%)	9/10 (90%)
Δ Stiffness (kPa)	0.83	≥0.11	4/4 (100%)	4/6 (67%)	8/10 (80%)
Cs (m/s)	0.79	≥1.73	3/4 (75%)	5/6 (83%)	8/10 (80%)
Stiffness (kPa)	0.75	≥3.16	2/4 (50%)	6/6 (100%)	8/10 (80%)
Wave attenuation (1/m)	0.58	≤44.35	3/4 (75%)	4/6 (67%)	7/10 (70%)

Post hoc ROC analysis in the subgroup of 10 patients with equivocal or overlapping imaging features (4 FNH, 6 HA). Thresholds were optimized using Youden’s J statistic within the subgroup. FNH, focal nodular hyperplasia; HA, hepatocellular adenoma.

## Data Availability

Data generated or analyzed during the study are available from the corresponding author by request. Anonymized participant raw data regarding MRE and the necessary software to process the MRE data are available after signing a data access agreement with the host institution (University Hospital Frankfurt) and INSERM.

## Supplementary Materials

The following supporting information can be downloaded at https://www.mdpi.com/article/10.3390/diagnostics16101569/s1. Table S1: Parameters of the gravitational 3D-MRE protocol.

## Abbreviations

The following abbreviations are used in this manuscript:

AUC	Area under the receiver operating characteristic curve
CI	Confidence interval
Cs	Shear wave speed
FNH	Focal nodular hyperplasia
FOV	Field of view
GAM	Generalized additive model
HA	Hepatic adenoma
HBP	Hepatobiliary phase
HCA	Hepatocellular adenoma
HCC	Hepatocellular carcinoma
ICC	Intraclass correlation coefficient
IHCA	Inflammatory hepatocellular adenoma
IQR	Interquartile range
MRE	Magnetic resonance elastography
MRI	Magnetic resonance imaging
OR	Odds ratio
PACS	Picture Archiving and Communication System
pSWE	Point shear wave elastography
ROC	Receiver operating characteristic
ROI	Region of interest
SD	Standard deviation
SWE	Shear wave elastography
TE	Echo time
TR	Repetition time
VIBE	Volumetric interpolated breath-hold examination
