# Supplementary material for: Gravitational 3D Magnetic Resonance Elastography for Differentiating Focal Nodular Hyperplasia and Hepatic Adenoma
Source: Diagnostics (Basel). 2026 May 21;16(10):1569. doi: 10.3390/diagnostics16101569 (PMC13206071; doi:10.3390/diagnostics16101569)
Supplement: Supplementary file 1 [file diagnostics-16-01569-s001.zip › diagnostics-4232111-supplementary.pdf]

Supplemental Material

Detailed gravitational 3D-MRE protocol

	**Gravitational 3D-MRE**
Sequence	Gradient-echo
Acquisition matrix (pixels)	96x78
Frequency (Hz)	60
TE (ms)	9.53
TR (ms)	104.25
Flip angle (degree)	25
No. of breath holds (n), breath hold time (s)	4, 14
FOV (mm)	384
No. of slices (n), slice thickness (mm)	8, 4
Parallel imaging acceleration factor	2
Phase offsets	4
Receiver bandwidth (Hz/pixel)	400
% FOV in phase-encoding direction	81,3
Motion encoding directions	M/P/S/Reference

Abbreviations: FOV, field of view; M, motion encoding in the measurement (readout) direction; MRE, magnetic resonance elastography; P, motion encoding in the phase-encoding direction; Reference, acquisition without motion-encoding gradient; S, motion encoding in the slice-select direction; TE, echo time; TR, repetition time.

Reconstruction of complex-valued shear modulus

The 3D-MRE reconstruction yields the full complex shear modulus $G^* = G' + iG''$ from the curl of the measured displacement field, thereby providing stiffness ($|G^*|$), attenuation $\alpha = \Im\left(\sqrt{\frac{\rho\omega^2}{G^*}}\right)$, and phase angle $Y = \frac{2}{\pi} atan\left(\frac{G''}{G'}\right)$ without any assumptions about rheological model or frequency dependence.

In the present study, MRE raw data were temporally filtered at the driving carrier frequency to extract, at each voxel, the complex-valued three-dimensional displacement vector. The real and imaginary parts were then individually smoothed in space using a three-dimensional Gaussian filter with a standard deviation of 0.75 pixels and a kernel size of 3×3×3 pixels. Spatial derivatives were computed in Fourier space.

To avoid ringing artifacts caused by the spatial mask delineating the region where waves are present, the data were embedded into a 128^3-pixel cube and tempered by solving the Laplace equation ($\nabla^2 U = 0$) in the outer domain, with the cube edges set to zero to ensure a smooth decay of the solution. An 11th-order Blackman–Harris filter was applied in the Fourier domain to effectively suppress high-frequency noise during derivative computation.

Finally, by applying the curl operator to the displacement field, the wave equation was transformed into a Helmholtz equation for the complex-valued three-dimensional curl. This equation was then solved using a minimum-χ^2 approach to obtain a single complex-valued shear modulus G^*.
